# Modulation of ion channels and transporters by carbon monoxide: causes for concern?

**DOI:** 10.3389/fphys.2012.00477

**Published:** 2012-12-20

**Authors:** Chris Peers

**Affiliations:** Faculty of Medicine and Health, University of LeedsClarendon Way, Leeds, UK

## Introduction

Heme oxygenases, particularly the inducible heme oxygenase-1 (HO-1), are the subject of intensive research since they show great promise as cytoprotective agents. These enzymes degrade heme to generate biliverdin, free iron, and carbon monoxide (CO), and this reaction appears to be crucial in a number of diverse biological systems. Decreasing free heme in itself is beneficial [for example in sepsis and infections (Gozzelino et al., 2010)], but much attention is also paid to the products of heme degradation as biologically active agents with therapeutic potential (Motterlini and Otterbein, 2010; Wegiel et al., 2012). Indeed, the physiological roles and potential of CO in particular (applied either by inhalation, or via CO releasing molecules; CORMs) are currently topics of intense research, with clinical trials currently evaluating its safety in human subjects and its usefulness in treating a variety of disorders (http://clinicaltrials.gov/ct2/search, *“carbon monoxide”*) (Wu and Wang, 2005; Durante et al., 2006; Kim et al., 2006; Ryter et al., 2006).

In this current climate of hopeful promise for CO-based therapies, it is easy to lose sight of the fact that it is a highly toxic gas: CO poisoning accounts for more than 50% of all fatal poisonings worldwide (Meredith and Vale, 1988; Cobb and Etzel, 1991; Varon et al., 1999). Although the number of fatalities arising from acute exposure may be considered relatively low, chronic exposure can much more commonly produce neurological and cardiovascular damage (Von Burg, 1999; Gandini et al., 2001; Omaye, 2002; Prockop and Chichkova, 2007), particularly in the aging population, and symptoms are often difficult to diagnose (Harper and Croft-Baker, 2004). Appropriately, therefore, much caution is taken as clinical trials progress and as our awareness of the biological actions of CO continues to develop.

**Figure 1:**
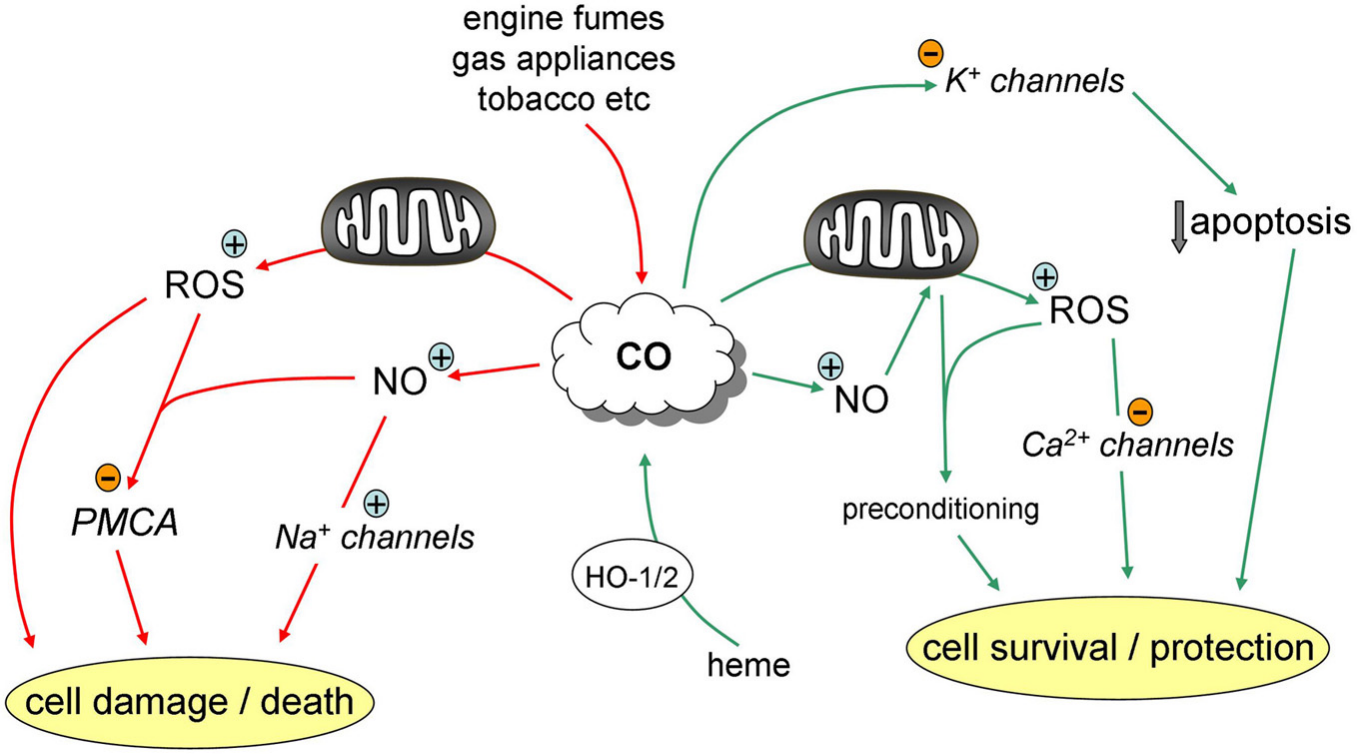
**Pathways contributing to the protective (green) and deleterious (red) effects of CO**.

In recent years, ion channels (and, more recently, transporters) have emerged as major targets for modulation by CO (Peers, 2011; Wilkinson and Kemp, 2011; Peers and Steele, 2012). Intriguingly, modulation of some channels by CO may contribute to its beneficial actions, yet the sensitivity of other channels to CO may account, at least in part, for some of its deleterious actions (summarized in Figure 1). In this article, I draw upon some recent examples of ion channel/transporter modulation by CO in the cardiovascular and central nervous systems in order to compare the beneficial and deleterious cellular effects of CO, and to examine whether we should be concerned about the therapeutic index of CO.

## Cardiovascular effects of CO

In the cardiovascular system, HO-1 (most commonly via its production of CO) exerts multiple beneficial effects. In addition to its known antihypertensive actions, it appears to be involved in numerous vascular diseases. Paradoxically, it suppresses vascular smooth muscle proliferation, yet augments endothelial proliferation, both of which can be considered beneficial. Suppression of smooth muscle proliferation is important in combating development of vessel thickening associated with vascular injury and grafting, and also in the progression of atherosclerosis [reviewed by Barbagallo et al. (2012)]. Stimulation of endothelial proliferation is dependent on vascular endothelial growth factor (VEGF) production, and is required for angiogenesis: HO-1 induction or CO exposure promotes endothelial VEGF production, proliferation, migration, and neovascularization (Jozkowicz et al., 2003).

In the heart and coronary vasculature, HO-1 expression can be increased by various stress factors including myocardial infarction or ischemia/reperfusion (I/R) (Lakkisto et al., 2002). The importance of HO-1 is well illustrated by studies employing transgenic mice: for example, in heterozygote HO-1^+/−^ KO mice I/R caused significantly greater cardiac damage than wild-type mice (Yoshida et al., 2001). In HO-1^−/−^ mice, chronic hypoxia (which normally up-regulates HO-1) caused significantly greater right ventricular hypertrophy, oxidative damage, and pulmonary hypertension (Yet et al., 1999). Furthermore, cardiac-specific overexpression of HO-1 strongly protects against I/R damage (Yet et al., 2001) and in a coronary ligation model of heart failure (Wang et al., 2010).

Although HO-1 is clearly protective, the relative contributions of heme reduction or biliverdin, Fe^2+^ and CO production are not fully elucidated. However, there is much evidence to support a key role for CO in HO-1-mediated cardioprotection. For example, Wang et al. (2010) reported that CORM-3 protected the myocardium against adverse remodeling following coronary ligation to a degree comparable with over-expression of HO-1. Indeed, CORMs have provided much support for the idea that HO-1 is cardioprotective via CO production. For example, infusion of CORM-3 during the reperfusion phase of I/R challenges reduced myocardial damage *in vitro* and *in vivo* (Clark et al., 2003; Guo et al., 2004), and CORM-3 can have a positive inotropic effect (Musameh et al., 2006).

As part of a study aimed at identifying potential mechanisms accounting for the protective effects of CO in the myocardium, we discovered that CO could inhibit the L-type Ca^2+^ current (Scragg et al., 2008). This effect was mediated by a CO-induced increase in ROS production from mitochondria, which modulated the channel via key cysteine residues located in the intracellular C-terminal domain of the α subunit. Inhibition of L-type Ca^2+^ channels would be predicted to reduce the energy demands of the myocardium, and hence could be considered as protective. However, when we subsequently examined the effects of CO and CO donors on single cardiac myocytes, the dominant effect was for CO to be pro-arrhythmic (Dallas et al., 2012): early after-depolarization events were detected via patch-clamp and Ca^2+^ imaging. These effects were attributable to CO-induced increases in NO levels, which led to an augmentation of the late component of the Na^+^ current via direct nitrosylation of Nav1.5. Crucially, rats exposed to 500 ppm CO displayed ECG abnormalities consistent with these effects, and when they were exposed to CO following isoprenaline injection, ventricular fibrillation, and death was observed. All effects were reversed by ranolazine, a known inhibitor of the late Na^+^ current (Dallas et al., 2012).

## Neurological effects of CO

The protective effects of CO in the central nervous system have also been demonstrated in a number of model systems. For example, CO inhalation (up to 250 ppm) provides clear beneficial effects against the damage of I/R brain injury and ischemic stroke (Wang et al., 2011; Zeynalov and Dore, 2009). Furthermore, pre-exposure to CO (also at 250 ppm) provided pre-conditioning protection against neurological damage (specifically, neuronal apoptosis) in a pig model of deep hypothermic circulatory arrest, which is a widely used technique in surgical treatment of heart defects (Mahan et al., 2012).

At the cellular level, pretreatment of primary cultures of cerebellar granule neurons with 250 ppm CO provided protection against apoptosis induced by oxidative stress or excitotoxic levels of exogenous glutamate (Vieira et al., 2008). Interestingly, protection appeared to be comparable to the pre-conditioning associated with sub-lethal ischemia, where neurons become more resistant to otherwise lethal ischemic challenges by prior exposure to a less severe challenge. Vieira et al. reported that protection afforded by CO involved activation of guanylate cyclase and subsequent activation of mitochondrial ATP-sensitive K^+^ channels. Importantly, they also reported increased intracellular ROS levels and stimulation of NO formation in response to CO exposure (Vieira et al., 2008). The increase in ROS formation is a key pre-conditioning step, since it triggers up-regulation of protective protein expression, including HO-1.

We subsequently provided another mechanism to account for neuroprotective effects of CO. According to a number of studies from Aizenman and colleagues (Redman et al., 2007; Pal et al., 2003, 2006), a key early stage in stress-induced neuronal apoptosis is the rapid insertion into the plasma membrane of K^+^ channels, particularly Kv2.1, which leads to loss of cytoplasmic K^+^ and the initiation of apoptotic signaling. Consistent with this hypothesis, we found that over-expression of Kv2.1 in HEK293 cells increased susceptibility to oxidative apoptotic stimuli, but that this could be prevented by CO i.e., CO provided protection against apoptosis specifically in Kv2.1 expressing cells (Dallas et al., 2011). Furthermore, CO directly inhibited Kv2.1 channels, and the currents arising from the “surge” of K^+^ channels in response to apoptotic stimuli. Importantly, these effects were reproduced in primary cultures of hippocampal neurons (Dallas et al., 2011). Interestingly, a recent study demonstrated that the CO donor, CORM-3, provided a degree of neuroprotection against a collagenase injection model of hemorrhagic stroke if administered in advance, but under other circumstances could exacerbate the associated damage (Yabluchanskiy et al., 2012).

The above described data indicate that CO may provide neuroprotection via multiple mechanisms. However, stimulation of both ROS and NO formation (implicated in some protective pathways) is not without risk, in part because it can lead to formation of the highly damaging ROS species peroxynitrite (ONOO^−^). Indeed, we reported recently that such effects of CO may account for some of the deleterious neurological effects of CO poisoning: we monitored Ca^2+^ homeostasis in human neuroblastoma (SH-SY5Y) cells and noted that exposure of cells to the CO donor CORM-2 caused an apparent increase in both voltage gated Ca^2+^ entry and prolonged receptor-mediated rises of [Ca^2+^]_i_ arising from mobilization of Ca^2+^ from endoplasmic reticulum stores and subsequent capacitative Ca^2+^ entry. These potentially deleterious effects of CO were abolished either by an antioxidant or by inhibition of NO formation with L-NAME (Hettiarachchi et al., 2012). NO donors alone were unable to mimic these actions of CO, indicating that both NO and ROS were required for CO to disrupt Ca^2+^ signaling. A rise of ONOO^−^ levels was detected via APF fluorescence, and the ONOO^−^ scavenger FeTPPs also inhibited the effects of CO. Mechanistically, we reasoned that for CO to disrupt so many Ca^2+^ signaling pathways it most likely acted via modulation of a common target protein, and we identified a CO-dependent down regulation of the plasmalemmal Ca^2+^ ATPase (PMCA) in both SH-SY5Y cells and also in whole brain homogenates from rats exposed to 1000 or 3000 ppm CO for 40 mins (Hettiarachchi et al., 2012).

## A question of concentration?

The example studies cited here raise the question of whether beneficial effects of CO differ from deleterious effects (shown schematically in Figure 1) simply because of concentration, or for some other, unidentified reasons. This is not a straightforward issue to resolve from the current literature, since CO is applied by various means (inhalation, or via CORMs), and final concentrations at the intended sites of action are difficult or impossible to determine accurately. Although the potential for neurological damage arising from loss of the PMCA was observed only at high (>1000 ppm) levels of inhaled CO *in vivo*, the cellular effects on PMCA were manifest using CORM-2 at 10–30 μM. Such concentrations are comparable to other reported effects of CO/CORMs which have been regarded, rightly or wrongly, as beneficial/physiological effects. Most electrophysiological studies have employed CORMs at concentrations of 1–100 μM (Peers, 2011; Wilkinson and Kemp, 2011), often at 30 μM or lower. Perhaps most alarmingly, the pro-arrhythmic actions of CO mediated by induction of the late Na^+^ current (Dallas et al., 2012) were observed when animals inhaled 500 ppm (or cells were exposed to 20–30 μM CORM-2 or -3). Such levels have been reported in heavy traffic or as a consequence of exposure to cigarette smoke (Reboul et al., 2012), and clinical trials are under way employing 250 ppm (see earlier). Clearly, as discussed by Reboul et al. (2012), the duration of exposure to CO is critical in determining whether its outcome is beneficial or detrimental, but as new targets for modulation become realized, it is becoming clear that a better understanding (or, better, a means of determination) of CO concentration at its sites of action is needed before we can describe effects of CO observed *in vitro* as potentially beneficial or detrimental. Such information is important in the progression of CO therapy.
